# Paternity and Dominance Loss in Male Breeders: The Cost of Helpers in a Cooperatively Breeding Mammal

**DOI:** 10.1371/journal.pone.0029508

**Published:** 2012-01-17

**Authors:** Sophie Lardy, Aurélie Cohas, Emmanuel Desouhant, Marion Tafani, Dominique Allainé

**Affiliations:** UMR CNRS 5558 “Biométrie et Biologie Evolutive”, Université de Lyon, Université Claude Bernard Lyon 1, Villeurbanne, France; University of Bristol, United Kingdom

## Abstract

Paternity insurance and dominance tenure length are two important components of male reproductive success, particularly in species where reproduction is highly skewed towards a few individuals. Identifying the factors affecting these two components is crucial to better understand the pattern of variation in reproductive success among males. In social species, the social context (*i.e.* group size and composition) is likely to influence the ability of males to secure dominance and to monopolize reproduction. Most studies have analyzed the factors affecting paternity insurance and dominance tenure separately. We use a long term data set on Alpine marmots to investigate the effect of the number of subordinate males on both paternity insurance and tenure of dominant males. We show that individuals which are unable to monopolize reproduction in their family groups in the presence of many subordinate males are likely to lose dominance the following year. We also report that dominant males lose body mass in the year they lose both paternity and dominance. Our results suggest that controlling many subordinate males is energetically costly for dominant males, and those unable to support this cost lose the control over both reproduction and dominance. A large number of subordinate males in social groups is therefore costly for dominant males in terms of fitness.

## Introduction

The number of offspring sired per year and the length of reproductive life are two major components of lifetime reproductive success [1]. In species living in social groups, particularly cooperative breeders, reproduction is often highly skewed towards a few dominant individuals that monopolize reproduction by suppressing reproductive functions, preventing access to potential mates or killing offspring of their subordinates [2]. In these species, the reproductive success of dominant males depends largely on paternity insurance at each reproductive event and on dominance tenure length. Determining the factors affecting these components is thus fundamental to understand the variation in reproductive success among males; and consequently the intensity of natural and sexual selection in social species [1].

The social context which dominant males have to cope with may be a key element of reproductive success. Subordinates within and outside the social unit compete with dominants both to reproduce and to reach dominance [3], [4] and their number may vary considerably [5], [6]. The size and the composition of social groups may determine the intensity of male-male competition. Since the control over their subordinates by dominant males is likely to decrease as the number of the subordinates increases (“limited control” hypothesis [7], [8]), dominants are expected to lose paternity when facing a large number of male subordinates. Similarly, dominants could be expected to lose their social status too in such a social context.

To date, most studies have analyzed factors affecting paternity insurance and dominance tenure separately. As expected, dominants do lose paternity when they are confronted by a large number of subordinate males, for example in meerkats *Suricata suricatta* [9], Savannah baboons *Papio cynocephalus* [10] and in Alpine marmots *Marmota marmota* [6], [11]. A few studies have shown that dominants are indeed more likely to lose dominance under such social conditions, for example in mandrills *Mandrillus sphinx* [12]. It is likely that dominant males can monopolize reproduction and also maintain their dominant status over long periods only in social conditions where competition is weak (*i.e.* few subordinates), while males facing highly competitive situations (*i.e.* a large number of subordinates) should be unable to insure paternity and should lose their dominant status rapidly. In other words, the males which lose control over reproduction, due to challenging social conditions, will also lose dominance in their social group rather rapidly.

The mechanisms by which the number of subordinate males reduces the ability of the dominant male to secure paternity and to maintain dominance are not well understood. One possible explanation is that it may be energetically costly for dominant males to control potential competitors present in the social unit [13]. Body mass (or body condition) has been shown to be a key determinant of the outcome of intra-sexual competition in several mammals [14]–[16] such as the ability of a male to both monopolize reproduction and maintain dominance over time [1]. Dominant males with low body mass may not be in adequate physical condition to prevent subordinates of the group, or external individuals, from getting fertilizations and even evicting them. Consequently, the number of subordinate males may affect body mass, which in turn affects paternity and dominance.

The Alpine marmot is a mammalian cooperative breeder, socially monogamous, which lives in family groups of 2 to 14 individuals comprising a dominant reproductive pair, mature and immature subordinates of both sexes and pups of the year [17]. Usually, dominant individuals monopolize reproduction by physiologically suppressing reproductive functions of subordinates of the same sex [18], [19]. Dominant females monopolize reproduction effectively (only two cases of reproduction by a subordinate female over 408 events of reproduction in our population). In contrast, dominant males frequently lose paternity, generally to transient males or in rare cases to subordinates of the group (unrelated to the dominant female) [6], [20].

Here we use a 18-year data set to (1) examine the effect of the number of potential competitors on the probability of losing both paternity and dominance in the male Alpine marmot; (2) test the prediction that males unable to monopolize reproduction are also unable to maintain their dominant status over time. We examine whether the probability of losing dominance is correlated positively with the occurrence of extra pair paternity (EPP) in the previous reproductive event. Finally, we attempt to identify the underlying mechanisms by investigating the link between the number of subordinates and the body mass of the dominant animals. We thus (3) test the prediction that the number of potential competitors influences dominants' body mass; and (4) examine whether the dominant male body mass influences its probability of losing dominance.

## Results

### Influence of the number of sexually mature male subordinates in the group on dominance tenure and on monopolization of reproduction

**Figure 1:**
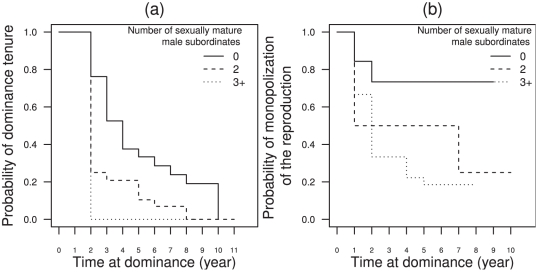
Kaplan-Meier plots showing the effect of the number of sexually mature male subordinates in the social group on (a) the probability that a male retains dominance, and (b) the probability that a male monopolizes matings. Three levels of the number of male subordinates are represented: none (0), medium (2) and high (3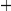).

**Figure 2:**
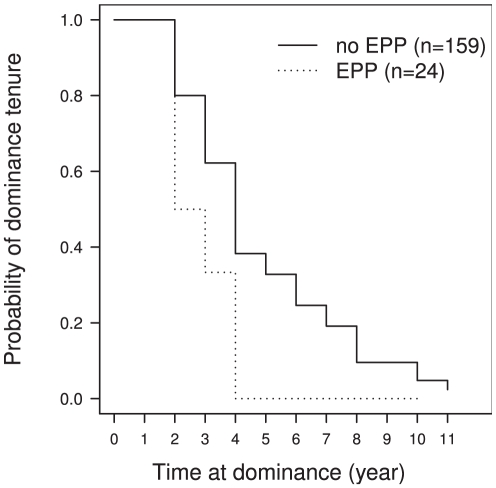
Kaplan-Meier plot showing the impact of extra-pair paternity at the previous event of reproduction on the probability that a male retains dominance. **The full line** represent survival curve where **no extra-pair paternity** occurred at the previous event of reproduction was observed while **the dotted line** represent survival curve where **extra-pair paternity** occurred at the previous event of reproduction.

Both the probability of maintaining dominance (Figure 1a) and the probability of monopolizing reproduction over time (Figure 1b) decrease as the number of sexually mature male subordinates present in the group increases. If the number of sexually mature subordinates in a group increases by one individual, the probability of losing dominance is multiplied by 1.27 [CI95%: 1.02–1.58] (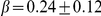, 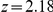, 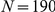 observations including 62 males, 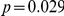, Figure 2a), and the probability that EPP occurs is multiplied by 1.36 [CI95%: 1.04–1.77] (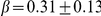, 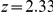, 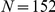 observations including 61 males, 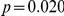, Figure 2b).

### EPP occurrence and dominance tenure

The likelihood that the dominant male would lose its dominance is multiplied by 2.16 [CI95%: 1.23–3.79] when an EPP occurred at the previous reproductive event (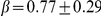, 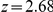, 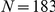 observations including 67 males, 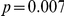, Figure 2). This suggests, as expected, that males unable to monopolize reproduction are not able to maintain their dominant position.

### A possible mechanism: male body mass and dominance

**Figure 3:**
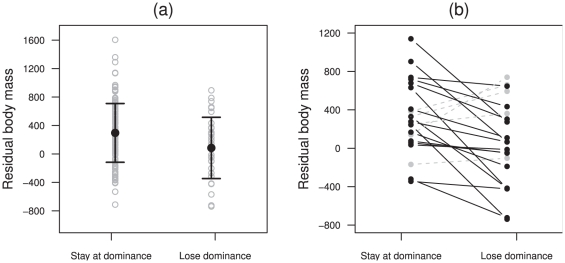
Comparison of the competitive abilities of males retaining dominance and males losing dominance. (a) The grey circles represent the observed residual body masses. The black dots represent the means surrounded by their standard deviation. (b) Comparison of competitive abilities of a given male the year it lost dominance and the years it was dominant. Males having a lower residual body mass the year of dominance loss are represented in black. Males having a higher residual body mass the year of dominance loss are represented in grey.

**Figure 4:**
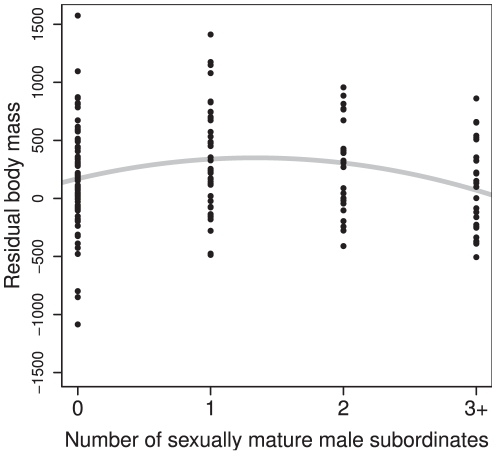
Impact of the number of sexually mature male subordinates present in a family group on the residual body mass of the dominant male. The dots represent the observed data, and the grey line represents the fitted model.

As expected, the probability of losing dominance increases as residual body mass (RBM) declines (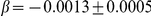, 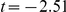, 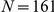 observations including 66 males, 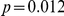). Dominant males which maintain dominance from one year to the next are 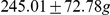 heavier, on average, than dominant males that lost dominance (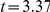, 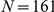 observations including 66 males, 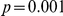, Figure 3a). Overall, a dominant male is lighter by 261.39 g [CI95%: 62.24–460.54] in the year it lost dominance compared to the years before (paired t-test: 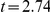, 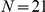, 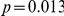, Figure 3b). This loss of body reserves represents up to 10% of their mass. Finally, the number of sexually mature male subordinates is related to the RBM of dominants. The RBM of dominant males is low when no male subordinate is present in the group, it increases when one subordinate male is present and then decreases when more than one male subordinate are present (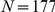 observations including 67 males, 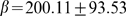, 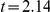, 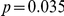, 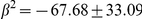, 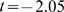, 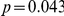, Figure 4). The RBM of the dominant females does not depend on the number of male subordinates (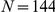 observations including 45 females, linear effect: 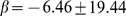, 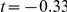, 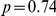, quadratic effect: 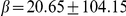, 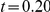, 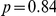, 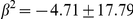, 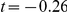, 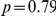).

## Discussion

The presence of several sexually mature males within family groups as well as the high reproductive skew suggest that, in the Alpine marmot, dominant males compete strongly with their subordinates for reproduction and dominant status. We show here that the number of male subordinates has, indeed, a strong effect on the ability of dominant males to monopolize reproduction. Specifically, the risk of paternity loss for dominants increases with the number of male subordinates, suggesting that dominant males lose the control over reproduction when facing several competitors. The role of male-male competition in paternity loss may have been underestimated in favour of female choice [21]–[23]. However, recently, Cohas and Allainé [24] have pointed out that, among monogamous species, family living species exhibit higher EPP rates suggesting that living with potential competitors enhances the likelihood of losing paternities. Our results strongly support this idea.

The observed pattern is consistent with the “limited control” hypothesis which implies that dominant males cannot control all the reproduction of the group despite their efforts to do so [7], [8]. This hypothesis seems to hold also in other social species (primates [10], [25], [26]; carnivores [27], [28]; birds [29]; fishes [30], [31]). We also show that the number of male subordinates in the social group greatly affects dominance tenure. Specifically, the number of male subordinates in the social unit decreases the ability of dominant males to maintain dominance and consequently reduces the length of time they remain in residence. The results presented here therefore evidence that the strong male-male competition for reproduction and dominance in family groups with numerous subordinate males does result in dominant males being less able to insure paternity and to maintain dominance over time. A negative effect of the number of subordinate males on the tenure of dominant males has been reported in some species [12] but not in all (meerkats [9]; lions *Panthera leo* [32]; Thomas' langur *Presbytis thomasi* [33]). The “limited control” hypothesis can thus be extended to cover dominance tenure.

We also report that those dominant males losing paternity are more prone to lose dominance the following year. This result suggests that, as male-male competition increases within the family group, dominant males lose control over reproduction and also over dominance. Virtually all dominant males which lost paternity had a dominance tenure 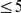 years, while dominant males able to secure paternity had dominance tenures as long as 11 years. A possible alternative explanation, proposed for monogamous birds, is that seeking extra-pair copulations is a strategy used by females to sample available mates before changing for a better partner (“mate sampling hypothesis”, [34]). Females are then expected to divorce and to re-pair with one of the extra-pair mates. The “mate sampling” hypothesis is unlikely to explain the observed association between paternity loss and dominance tenure in Alpine marmots for two reasons. First, in year-round territorial and long-lived monogamous species, mate switching resulting from female choice is unlikely to occur due to the high cost associated with the lost of the territory [35]. Secondly, in the population studied here, the extra-pair mate became the new dominant in only 3 out of the 25 cases where the dominant male had lost paternity. We conclude that numerous subordinate males may be costly for dominant males since they limit the dominants' control over both reproduction and dominance.

How do social factors affect the ability of dominant males to monopolize reproduction and secure dominance? One explanation is that it is simply impossible for dominant males to control each of numerous subordinates with fighting abilities [36]. Mate guarding may thus be less effective as the number of challengers in the social group increases. In the presence of many competitors, it will be more and more difficult to prevent challengers, from within or outside the social group, to access the female. Another possible explanation is that the control of subordinates is costly for dominants [7] and controlling a large number of potential competitors can have a high energetic cost [37]. Body mass is an important determinant of the ability of males to maintain dominance in Alpine marmots: males losing dominance are lighter than those which retain dominance from one year to the next, and loss of dominance is associated with mass loss (5–10%). This pattern has been found in most mammals [14], [16]. The fact that the number of subordinate males in the social unit affects dominant male body mass negatively indicates that it may indeed be energetically costly for dominant males to control a high number of potential competitor in the social unit (see also [13] for a similar example in the cichlid fish *Neolamprologus pulcher*). A male with a large number of male subordinates is in lower body condition and may consequently be more likely to lose paternity and then dominance.

We suggest that the general process by which the number of subordinates imposes costs on the dominants is that controlling many subordinate males leads to an energetic cost for these males, and this leads to losing body reserves. This mass loss may in turn lower their capacity to guard their mate effectively, thus allowing other males to gain extra-pair paternity and lowering the dominant male's ability to win contests for dominance. The cost generated by the presence of competitors in social groups may counterbalance the benefits provided by social living [38] and dominants are then expected to make a trade-off between the costs and the benefits of having subordinate males in their family groups [11], [39]. Understanding the interplay between group composition and the reproductive success of individuals in social species thus represents a crucial point to identify the evolutionary forces shaping animal societies.

## Materials and Methods

### 0.1 Ethics Statement

The field work conducted in the Alpine marmots was undertaken after acceptance of the project by the scientific committee of the Vanoise National Park, and after the deliverance of the permit number AP n 2010/121 by the *Préfecture de la Savoie*. Sophie Lardy is authorized for experimentation with animals, issued by the French Ministry of Agriculture and Fisheries (diploma n/0ETRY20090520). French law does not demand approval by an ethical committee.

### 0.2 Study species

Alpine marmots are cooperatively breeding rodents which live in family groups where only the dominant pair reproduces, as a rule. Both males and females may stay as dominants on the same territory for several years (up to 11 and 14 years for males and females respectively on our study population), until they die or get evicted by a competitor. Eviction is generally followed by death, for dominants of both sexes [35]. Relatedness among family members is extremely high as virtually all individuals are offspring of at least one of the dominants. Male subordinates (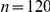) are related to the dominant male in 81% of cases and to the dominant female in 79% of cases. In only two cases were subordinates not certainly related to one of the dominants: i) when EPP occurred, extra-pair pups are unrelated to the dominant male, ii) when a new dominant male or female arrives in the territory. In general, when a new individual establishes itself as dominant, the same sex individuals that were subordinates in this group leave the group and pups are killed, which reduces the number of unrelated individuals of the same sex in the family groups. Individuals reach sexual maturity when two years old. They may delay dispersal and stay in the family group as subordinates, and reach dominance in their natal territory (20% of the dominant males) or may disperse in search of a dominant position (80% of the dominant males). Individuals never join groups as subordinates. Male subordinates are “helpers” since their presence during hibernation increases offspring survival [11], [17]. Subordinate males of the group are potential competitors of the dominant male as they may attempt to get fertilization from the dominant female if they are not related, or to evict the dominant male and take over the dominant position. When subordinate males reached dominance in its natal territory, their mother had previously been replaced as the dominant. We observed only one case of incest among 408 events of reproduction in this population.

### 0.3 Field methods and data collection

Data were collected in a wild population of Alpine marmots located in the nature reserve of La Grande Sassière (at 2340 m a.s.l., French Alps, 4529
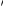
N, 659
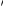
E) from 1990 to 2007. Over 80% of the marmots belonging to 24 family groups were caught at least once every year, between mid-April to mid-July, using two-door live traps baited with dandelions (*Taraxacum densleonis*). Once captured, the animals were tranquilized with Zolétil 100 (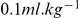), sexed, aged from their size up to 3 years of age, weighed and individually marked with a numbered ear-tag and a transponder. Social status was determined for all individuals from scrotal development for males and teat development for females. Group composition was determined from capture data and completed by daily observations of the families. We counted for each group, the number of adults, two-year-olds and juveniles of each sex. Every year, scent marking and aggressive behaviour noted during behavioural observations were also used to confirm dominance status [40].

### 0.4 Paternity analyses

Genetic analyses were performed on 16 microsatellites following Cohas *et al*'s. [41] protocol. Genotypes of each young and of the dominant pair were used to check maternity of the dominant female and paternity of the dominant male, using both exclusion of paternity and paternity analyses conducted with Cervus 3.0.3 software [42] (for details see Cohas *et al.* [41]). A young marmot was considered as a within-pair offspring if its genotype matched with the dominant male genotype, and as extra-pair if it did not. We noted an occurrence of extra-pair paternity (EPP) when at least one young of a litter was identified as extra-pair young.

### 0.5 Data analyses

#### Influence of the number of sexually mature male subordinates in the group on dominance tenure and on monopolization of reproduction

The influence of the average number of sexually mature male subordinates present between two reproductive seasons on the probability of retaining dominance and the probability of monopolizing reproduction, were analyzed using Cox's proportional hazards model [43]. A Cox regression assumes that the probability per unit of time that a dominant male lose its dominant status (or hazard rate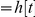), is the product of a baseline probability and a factor representing the joint effect of the covariates, with 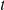 representing the time elapsed since the dominant male acquired the dominant status. In our population, male subordinates reached dominance at 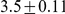 years old, thus leading to a high correlation between time and age. The comparison of dominance loss was done on individuals of roughly the same age. The 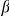 values express the contribution of each explanatory variable to the overall tendency to lose dominance. These coefficients are interpreted through the exponential term, the hazard ratio. A hazard ratio higher than unity indicates that the corresponding covariate has an increasing influence on the tendency of a male to lose its dominance, *i.e.* it reduces its dominance tenure. Conversely, a hazard ratio lower than one corresponds to an increase in its dominance period [44]. The same reasoning was applied for paternity loss. The repeated measures on same territories were taken into account in the model of dominance loss and the model of paternity loss. Regression coefficients were estimated by maximization of the partial likelihood (for details, see [45]).

#### EPP occurrence and dominance tenure

The influence of EPP occurrence on the probability to lose dominance the following year was also analyzed using Cox's proportional hazards model with occurrence of EPP encoded as a binomial variable and entered in the model as a time-dependent covariate [46].

#### A possible mechanism: male body mass and dominance

Body mass in marmots varies with the seasons, so body mass was corrected using linear models including the date of capture, its quadratic term and year. The residuals (RBM for residual body mass) were used thereafter. An additional correction for body size (residual body condition) did not change the results, so we present only the results with RBM. The influence of RBM on the probability of losing dominance was investigated first. Generalized mixed models (GLMM) with male identity within territory as random factors, a logit link function and a binomial error distribution were used to account for repeated measures and for the binomial distribution of the dependent variable. To verify that the effect of the number of subordinates on male RBM was not an effect of resource limitation due to high densities of individuals in the territories, the effect of the number of subordinates on dominant female RBM was also studied with the same procedure as the one used for males. The RBM of males staying dominant was then compared to the RBM of males losing dominance using linear mixed models with male identity within territory as random factors to account for repeated measures. Finally we compared the RBM of a male (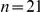) the year it lost dominance with its RBM in the years it stayed dominant, using a paired t-test. The influence of the number of sexually mature male subordinates on the RBM of dominant males was investigated using linear mixed models with male identity within territory as random factors to account for repeated measures.

Statistical analyses were performed with R 2.10.1 [47] using the function lme in the “MASS” library for linear mixed models, the function glmer in the “lme4” library [48] for the GLMM, the function coxph in the “survival” library [49] for the Cox's proportional hazards model. The level of significance is set to 0.05 and parameter estimates are given 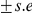.

## References

[1] Clutton-Brock TH (1988). Reproductive success: studies of individual variation in contrasting breeding systems.

[2] Clutton-Brock TH, Hodge SJ, Flower TP, Spong GF, Young AJ (2010). Adaptive suppression of subordinate reproduction in cooperative mammals.. Am Nat.

[3] Emlen S (1982). The evolution of helping. II. The role of behavioral conict.. Am Nat.

[4] Clutton-Brock TH (2009). Structure and function in mammalian societies.. Phil Trans R Soc Lond B.

[5] Komdeur J (2001). Mate guarding in the Seychelles warbler is energetically costly and adjusted to paternity risk.. Proc R Soc B.

[6] Cohas A, Yoccoz NG, Silva A, Goossens B, Allainé D (2006). Extra-pair paternity in the monog amous Alpine marmot (*Marmota marmota*): the roles of social setting and female mate choice.. Behav Ecol Sociobiol.

[7] Clutton-Brock TH (1998). Reproductive skew, concessions and limited control.. Trends Ecol Evol.

[8] Reeve HK, Emlen S, Keller L (1998). Reproductive sharing in animal societies: reproductive incen tives or incomplete control by dominant breeders?. Behav Ecol.

[9] Spong GF, Hodge SJ, Young AJ, Clutton-Brock TH (2008). Factors affecting the reproductive success of dominant male meerkats.. Mol Ecol.

[10] Alberts SC, Watts HE, Altmann J (2003). Queuing and queue-jumping: long-term patterns of reproductive skew in male savannah baboons, *Papio cynocephalus*.. Anim Behav.

[11] Allainé D, Theuriau F (2004). Is there an optimal number of helpers in Alpine marmot family groups?. Behav Ecol.

[12] Setchell J, Wickings E (2006). Life history in male mandrills (*Mandrillus sphinx*): physical devel opment, dominance rank, and group association.. Am J Phys Anthropol.

[13] Mitchell JS, Jutzeler E, Heg D, Taborsky M (2009). Gender differences in the costs that subordinate group members impose on dominant males in a cooperative breeder.. Ethology.

[14] Clutton-Brock TH, Guinness F, Albon S (1982). Red deer: behavior and ecology of two sexes.

[15] Haley M, Deutsch C, Le Boeuf B (1994). Size, dominance and copulatory success in male northern elephant seals, *Mirounga angustirostris*.. Anim Behav.

[16] Ellis L (1995). Dominance and reproductive success among non human animals: a cross-species comparison.. Ethol Sociobiol.

[17] Allainé D (2000). Sociality, mating system and reproductive skew in marmots: evidence and hy potheses.. Behav Process.

[18] Arnold W, Dittami J (1997). Reproductive suppression in male Alpine marmots.. Anim Behav.

[19] Hacklander K (2003). Reproductive suppression in female Alpine marmots, *Marmota marmota*.. Anim Behav.

[20] Goossens 302 B, Graziani L, Waits LP, Farand E, Magnolon S (1998). Extra-pair paternity in the monogamous Alpine marmot revealed by nuclear DNA microsatellite analysis.. Behav Ecol Sociobiol.

[21] Jennions MD, Petrie M (2000). Why do females mate multiply? A review of the genetic benefits.. Biol Rev Camb Philos Soc.

[22] Griffith SC, Owens IPF, Thuman KA (2002). Extra pair paternity in birds: a review of interspecific variation and adaptive function.. Mol Ecol.

[23] Westneat D, Stewart I (2003). Extra-pair paternity in birds: causes, correlates, and conict.. Annu Rev Ecol Syst.

[24] Cohas A, Allainé D (2009). Social structure inuences extra-pair paternity in socially monogamous mammals.. Biol Lett.

[25] Kutsukake N, Nunn CL (2006). Comparative tests of reproductive skew in male primates: the roles of demographic factors and incomplete control.. Behav Ecol Sociobiol.

[26] Widdig A, Bercovitch F, Streich W, Sauermann U, Nürnberg P (2004). A longitudinal analysis of reproductive skew in male rhesus macaques.. Proc R Soc B.

[27] Clutton-Brock TH, Brotherton PN, Russell aF, O'Riain MJ, Gaynor D (2001). Cooperation, control, and concession in meerkat groups.. Science.

[28] Cant MA (2000). Social control of reproduction in banded mongooses.. Anim Behav.

[29] Haydock J, Koenig WD (2003). Patterns of reproductive skew in the polygynandrous acorn wood pecker.. Am Nat.

[30] Awata S, Munehara H, Kohda M (2005). Social system and reproduction of helpers in a cooper atively breeding cichlid fish (*Julidochromis ornatus*) in lake Tanganyika: field observations and parentage analyses.. Behav Ecol Sociobiol.

[31] Heg D (2006). Cooperative breeding in the lake Tanganyika cichlid *Julidochromis ornatus*.. Environ Biol Fish.

[32] Packer C, 327 Herbst L, Pusey A, Bygott J, Hanby J (1988). Reproductive success: studies of individual variation in contrasting breeding systems.

[33] Steenbeek R (2000). Reproductive success: studies of individual variation in contrasting breeding systems.

[34] Heg D, Ens B, Burke T, Jenkins L (1993). Why does the typically monogamous oystercatcher (*Haematopus ostralegus*) engage in extra-pair copulations?. Behaviour.

[35] Lardy S, Cohas A, Figueroa I, Allainé D (2011). Mate change in a socially monogamous mammal: evidences support the “forced divorce” hypothesis.. Behav Ecol.

[36] Cowlishaw G, Dunbar RI (1991). Dominance rank and mating success in male primates.. Anim Behav.

[37] Rubenstein DR, Shen SF (2009). Reproductive conict and the costs of social status in cooperatively breeding vertebrates.. Am Nat.

[38] Sparkman AM, Adams J, Beyer A, Steury TD, Waits L (2011). Helper effects on pup lifetime fitness in the cooperatively breeding red wolf (*Canis rufus*).. Proc R Soc B.

[39] Young AJ, Carlson AA, Monfort SL, Russell AF, Bennett NC (2006). Stress and the sup pression of subordinate reproduction in cooperatively breeding meerkats.. Proc Natl Acad Sci U S A.

[40] Bel M, Porteret C, Coulon J (1995). Scent deposition by cheek rubbing in the Alpine marmot (*Marmota marmota*) in the French Alps.. Can J Zool.

[41] Cohas A, Yoccoz N, Bonenfant C, Goossens B, Genton C (2008). The genetic similarity between pair members inuences the frequency of extrapair paternity in Alpine marmots.. Anim Behav.

[42] Kalinowski ST, Taper ML, Marshall TC (2007). Revising how the computer program cervus ac commodates genotyping error increases success in paternity assignment.. Mol Ecol.

[43] Cox D 353 (1972). Regression models and life tables (with discussion).. J Roy Statist Soc Ser B.

[44] Wajnberg E (2006). Time allocation strategies in insect parasitoids: from ultimate predictions to proximate behavioral mechanisms.. Behav Ecol Sociobiol.

[45] Kalbeisch J, Prentice R (2002). The statistical analysis of failure time data, volume 5..

[46] Kleinbaum D, Klein M (2005). Survival analysis: a self-learning text.

[47] R Development Core Team (2010). R: a language and environment for statistical computing. R Foundation for Statistical Computing, Vienna, Austria.. http://www.R-project.org/.

[48] Bates D, Maechler M (2010). lme4: linear mixed-effects models using S4 classes.. http://CRAN.R-project.org/package=lme4.

[49] Therneau T, original Splus R port by Thomas Lumley (2011). survival: survival analysis, including penalised likelihood.. http://CRAN.R-project.org/package=survival.

